# Association of type D personality with vascular health in adolescents

**DOI:** 10.3389/fcvm.2025.1591008

**Published:** 2025-07-16

**Authors:** Cheng Fangman, Lv Han, Sheng Nan, Ge Binqian, Liu Ying, Zhang Chunmei, Zhou Ping, Zhu Fenfen, Shen Juan

**Affiliations:** School of Nursing, Suzhou Vocational Health College, Suzhou, China

**Keywords:** type d personality, negative affectivity, vascular elasticity, adolescents, sleep quality, health behaviors

## Abstract

**Objective:**

This study aimed to evaluate the association between type D personality and vascular health in adolescents.

**Methods:**

A total of 645 adolescents were involved in this study. All completed questionnaires assessing demographic and sociological characteristics, Type D personality Scale, Scale for Healthy Lifestyle of College Students (SHLCS), and Self-Rating Scale of Sleep (SRSS). Vascular health was evaluated using a fingertip sensor with biofeedback technology to measure vascular wall elasticity. The effect of type D personality was analyzed using both dichotomous and continuous methods.

**Results:**

When analyzed as a binary variable, type D personality significantly affected vascular health scores [*β* = −0.169, 95% confidence interval (CI): [−4.001 to −1.483], *P* < 0.001]. When treated as continuous variables, negative affectivity (NA) exhibited an independently negative association with vascular health [*β* = −0.240, 95% CI: (−0.569 to −0.049), *P* = 0.020], whereas social inhibition (SI) and the interaction between NA and SI did not demonstrate significant effects. Additionally, abdominal circumference [*β* = −0.171, 95% CI: (−0.198 to −0.039), *P* = 0.004], pulse rate [*β* = −0.093, 95% CI: (−0.127 to −0.010), *P* = 0.021], and SRSS score [*β* = −0.155, 95% CI: (−0.336 to −0.110), *P* < 0.001] were negatively associated with vascular health. Conversely, stress tolerance [*β* = 0.211, 95% CI: (0.062–0.139), *P* < 0.001], exercise behavior [*β* = 0.078, 95% CI: (0.001–0.192), *P* = 0.048], and stress management behavior [*β* = 0.226, 95% CI: (0.328–0.780), *P* < 0.001] were positively associated with better vascular health.

**Conclusion:**

The findings suggest that type D personality is associated with vascular health in adolescents. Moreover, the NA component of the type D, but not the SI and NA*SI interaction, may drive the connection between type D personality and vascular health in adolescents. These findings highlighted the importance of initiating cardiovascular and cerebrovascular health promotion and disease prevention strategies from childhood.

## Introduction

Vascular aging begins as early as childhood. The initial phase is characterized by arterial stiffening and a loss of elasticity, preceding structural degeneration and the development of atherosclerosis (1). A decline in arterial elasticity is one of the earliest indicators of vascular disease, occurring prior to observable anatomical changes in the vessel walls. Consequently, early assessment of vascular health in adolescents is critical to prevent the onset of cardiovascular disease (2). Additionally, it is crucial to identify the risk factors during adolescence for guiding preventive strategies. In addition to traditional physiological risk factors, psychological factors may also contribute to reduced arterial elasticity in this age group. One such factor is type D personality, or “distressed” personality, defined by the presence of both negative affectivity (NA) and social inhibition (SI). Cases with this personality type may experience persistent negative emotions, such as anxiety, tension, and sadness, and mainly suppress emotional expression in social settings. Type D personality has been identified as an independent risk factor for cardiovascular disease, potentially influencing its onset and progression through both biological and behavioral pathways (3, 4). Furthermore, the European Cardiovascular Prevention guidelines have emphasized type D personality since 2016 (5). However, recent research has questioned the validity of type D personality as a unitary construct, suggesting that its observed health effects may be primarily driven by one specific dimension, with the contributions of the other dimension being smaller or inconsistent (6, 7). Currently, it remains unclear whether the impact of type D personality on vascular health stems predominantly from the overall construct, its individual dimensions, or their interaction. To address this, the present study will examine the association between type D personality and vascular health in adolescents, utilizing both categorical and continuous variable approaches for the overall construct and its individual dimensions.

Cardiovascular disease, a psychosomatic disorder, is significantly influenced by behavioral factors that affect both its onset and progression. Previous research has demonstrated that lifestyle behaviors, such as engaging in regular physical activity and maintaining healthy dietary habits, can enhance the stability of coronary atherosclerotic plaques (8). Several studies have demonstrated that prolonged poor sleep quality can lead to sustained elevations in systemic blood pressure, resulting in the disruption and degradation of vascular elastic fibers, and ultimately a decrease in vascular elasticity (9, 10). Moreover, healthy individuals who engage in regular physical exercise generally exhibit better arterial elasticity than those who are sedentary (11). Therefore, lifestyle factors may exert a notable impact on vascular elasticity during adolescence.

In this study, photoplethysmography was employed to evaluate vascular elasticity and explore its variations and related risk factors in adolescents exhibiting type D personality. The objective was to provide a practical basis for early evaluation and intervention targeting vascular elasticity changes in this population.

## Methods

### Study design and participants

This cross-sectional study recruited 680 college students, who aged 17–19 years, from Suzhou Health Vocational College (China). Participants were eligible for inclusion if they met the following criteria: (1) aged 16–19 and currently enrolled as nursing students; (2) no history of cardiovascular or metabolic diseases, with normal blood glucose levels as well as normal liver and kidney function; (3) no use of medications affecting vascular function within the past three months; and (4) provided written informed consent and agreed to participate for the duration of the study. Exclusion criteria were summarized as follows: (1) presence of mental illness or severe physical illness; and (2) history of smoking, heavy alcohol consumption, or recent surgery. Additionally, 18 participants were excluded because they completed the questionnaire in less than 300 s, indicating potentially insufficient engagement.

Participants were informed of the study's objectives and provided written informed consent prior to enrollment. They were then instructed to complete a series of questionnaires independently, in which demographic information was gathered, and type D personality, depression, sleep quality, and lifestyle behaviors were assessed. Arterial elasticity was measured using an intelligent pressure analyzer based on photoplethysmographic technology. Following the measurement, participants manually recorded the results in an online questionnaire platform (Questionnaire Star). The entire process took approximately 30 min per participant. This study has been approved by the ethics committee of the university (Approval No. AWYXLL202511).

### General demographic and sociological assessments

Basic information: Participants self-reported demographic and lifestyle information, including gender, age, grade level, family history of cardiovascular disease, smoking status, and alcohol consumption.

Physical fitness: Anthropometric and physiological measurements included height, weight, body mass index (BMI), abdominal circumference (AC), systolic blood pressure (SBP), diastolic blood pressure (DBP), and pulse rate. Height and AC were measured by participants in pairs using a tape measure. Weight was measured using a standard weight scale. Blood pressure (SBP and DBP) and pulse were assessed using a validated electronic blood pressure monitor. All measurements were taken in a quiet environment, and participants were in a relaxed and appropriate posture. Each measurement was conducted twice, with an interval of 30–60 s between readings, and the average of the two values was used for analysis. Pulse rate, defined as the rhythmic dilation of the arterial wall corresponding to cardiac contractions, served as an important index of cardiovascular function.

### Assessment of type D personality

Type D personality was measured by Type D Personality Scale (DS14) (12), including two dimensions, NA and SI, and each contains 7 items. Participants responded using a 5-point Likert scale ranging from 0 (“false”) to 4 (“true”). A score of ≥10 on both subscales indicated the presence of type D personality. The DS14 scale has demonstrated strong psychometric properties, with Cronbach's alpha coefficients of 0.92 for NA and 0.79 for SI, and test-retest reliability coefficients of 0.79 and 0.81, respectively, supporting its validity and reliability in Chinese adolescents (13). In this study, the Cronbach coefficients of NA and SI were 0.894 and 0.824, respectively.

### Assessment of healthy lifestyle

Adolescents' healthy lifestyle was assessed by the Evaluation Scale for Healthy Lifestyle of College Students (SHLCS), comprising 8 dimensions and 33 items. Each item was rated on a 5-point Likert scale, and scores of responses ranged from 1 (“never”) to 5 (“always”). The eight dimensions and their respective score ranges were summarized as follows: exercise behavior (3–15), regular lifestyle behavior (3–15), dietary and nutritional behavior (4–20), health hazard behavior (2–10), health responsibility behavior (5–25), interpersonal relationship behavior (6–30), stress management behavior (5–25), and life appreciation behavior (5–25). Higher scores indicate greater engagement in the corresponding behaviors. Factor loadings for each dimension were above 0.52, indicating good construct validity (14). In the present study, the Cronbach's alpha coefficient for each subscale ranged from 0.658 to 0.833.

### Assessment of sleep quality

The sleep quality was measured by the Self Rating Scale of Sleep (SRSS), developed by Professor Li Jianming (15), who is the Executive Director of the Chinese Psychological Health Association. The SRSS comprises 10 items, each rated on a 5-point scale, with total scores ranging from 10 to 50. Higher scores indicate more severe sleep disturbances. This scale is frequently utilized to assess sleep quality among college students. The SRSS has demonstrated adequate reliability, with the Cronbach coefficient of above 0.64 (16). The internal consistency of this scale was 0.789 in the present study.

### Vascular health in adolescents

This study utilized a non-invasive intelligent detection system to measure vascular health. The device, developed by Professor Wang Erdong's team at Soochow University, has been certified by the U.S. Food and Drug Administration (FDA). It employs photoplethysmographic (PPG) technology to monitor changes in blood flow at the fingertips. The system consists of finger-worn sensors and a Smart Pulse terminal, emitting light at specific wavelengths that penetrate the skin. The reflected or transmitted light signals, influenced by blood volume fluctuations, are converted into electrical signals to generate an accelerated pulse wave, from which vascular elasticity and overall vascular health can be inferred (17). During testing, the device is connected via Bluetooth to the *Heart Bar Stress Analysis* mobile application. The Smart Pulse sensor is placed on the participant's index finger, and each test lasts approximately 2 min. The resulting report provides detailed quantitative assessments, including physical stress, mental stress, stress resistance, arterial elasticity, peripheral vascular elasticity, overall vascular health, and the APG waveform analysis. Specifically, waveform component “a” refers to the reference waveform (normal range: 275 ± 15%), “b” represents the initial contraction wave, indicating cardiac output intensity (higher values are better), and “c” reflects the late contraction augmentation wave, indicative of vascular elasticity (higher values suggest better elasticity). The use of this technology realizes the measurement of vascular wall elasticity values using fingertip pulse wave signals. Its simplicity and affordability make it well-suited for large-scale vascular health screenings in the general population.

### Statistical analysis

The statistical analysis was carried out using SPSS software. Continuous variables following a normal distribution were expressed as mean ± standard deviation (SD), while non-normally distributed variables were presented as median and interquartile range (IQR). Categorical variables were summarized using frequencies and percentages. Group comparisons of normally distributed continuous variables were conducted using an independent-samples *t*-test, whereas the Mann–Whitney *U* test was applied for non-normally distributed data. Categorical variables were analyzed using the Chi-square (*χ*^2^) test. To examine the association between type D personality and vascular health in adolescents, multivariate analyses were conducted. Covariates were selected based on prior literature. Age and gender were included as standard control variables. Additional covariates included BMI, AC, SBP, DBP, pulse rate, stress index, stress tolerance, and scores for the following lifestyle factors, including exercise behavior, regular lifestyle behavior, dietary behavior, health hazard behavior, health responsibility behavior, interpersonal relationship behavior, stress management behavior, life appreciation behavior, and sleep quality (SRSS score). These variables were included to determine whether type D personality could serve as an independent predictor of vascular health. Statistical significance was defined as a two-sided *P* < 0.05.

## Results

### Baseline characteristics in type D personality and non-type D personality groups

The study included 645 participants, of whom 18.9% (*n* = 122) were male and 81.1% (*n* = 523) were female. Demographic characteristics and questionnaire data for all participants are presented in Tables 1, 2. Participants' mean age was 18.32 (SD = 0.65) years. The overall prevalence of type D personality in the sample was 28.84%. Notably, SBP, DBP, and pulse rate were significantly higher in participants with type D personality compared with those without (*P* < 0.05). Additionally, the prevalence of type D personality was significantly higher in men than that in women (*P* = 0.013). No other variables exhibited statistically significant differences between the type D and non-type D groups (Table 1).

**Table 1:** Participants' baseline characteristics in type D personality and non-type D personality groups.

Variables	Total (*n* = 645)	Type D (*n* = 186)	Non-type D (*n* = 459)	Test value	*P* value
Age, M (S.D.), years	18.32 (0.65)	18.29 (0.651)	18.34 (0.651)	0.798	0.425
Gender [male, *n* (%)]	122 (18.9)	98 (21.4%)	24 (12.9%)	6.159	0.013
Height, M (S.D.), cm	164.61 (6.37)	164.40 (6.48)	164.69 (6.33)	0.511	0.609
Weight, M (S.D.), kg	57.45 (10.69)	58.60 (11.46)	56.98 (10.34)	−1.678	0.094
BMI, M (S.D.), kg/m^2^	21.13 (3.44)	21.53 (3.48)	20.97 (3.41)	−1.859	0.063
AC, M (S.D.), cm	73.08 (10.62)	74.24 (12.51)	72.61 (9.73)	−1.594	0.112
SBP, M (S.D.), mmHg	104.30 (11.49)	106.03 (13.01)	103.60 (10.74)	−2.447	0.015
DBP, M (S.D.), mmHg	67.53 (8.61)	69.72 (9.00)	66.64 (8.29)	−4.031	<0.001
Pulse, M (S.D.), per min	82.04 (10.01)	86.41 (10.75)	80.27 (9.13)	−6.849	<0.001
Family history of CAD, *n* (%)	40 (6.2%)	24 (5.2%)	16 (8.6%)	2.589	0.108
Smoking, *n* (%)	20 (3.1%)	14 (3.1%)	6 (3.2%)	0.014	0.907
Alcohol, *n* (%)	18 (2.8%)	12 (2.6%)	6 (3.2%)	0.182	0.669

BMI, body mass index; AC, Abdominal circumference; SBP, systolic blood pressure; DBP, diastolic blood pressure; M (S.D.), mean (standard deviation).

**Table 2:** Participants' psychological state and healthy behaviors in type D personality and non-type D personality groups.

Variables	Total (*n* = 645)	Type D (*n* = 186)	Non-type D (*n* = 459)	Test value	*P* value
Physical stress, M (S.D.)	45.71 (6.31)	46.99 (4.15)	45.19 (6.94)	−3.306	0.001
Mental stress, M (S.D.)	47.99 (8.60)	48.09 (6.54)	47.95 (9.30)	−0.217	0.828
Stress index, M (S.D.)	49.90 (7.02)	50.70 (5.66)	49.58 (7.48)	−2.061	0.040
Stress tolerance, M (S.D.)	66.28 (15.49)	62.63 (16.73)	67.75 (14.72)	3.639	<0.001
SRSS, M (S.D.)	23.15 (5.13)	26.26 (5.41)	21.88 (4.43)	−9.787	<0.001
Healthy lifestyle, M (S.D.)					
Exercise behavior	8.81 (2.92)	7.89 (2.91)	9.19 (2.85)	5.209	<0.001
Regular lifestyle behavior	10.67 (2.58)	9.78 (2.65)	11.02 (2.46)	5.662	<0.001
Dietary behavior	13.98 (3.20)	13.05 (3.28)	14.36 (3.08)	4.793	<0.001
Health hazard behavior	2.42 (1.23)	2.43 (0.97)	2.41 (1.33)	−0.191	0.848
Health responsibility behavior	20.65 (3.46)	19.26 (4.39)	21.22 (2.81)	5.627	<0.001
Interpersonal relationship behavior	23.31 (4.56)	22.90 (5.49)	24.48 (4.11)	1.314	0.190
Stress management behavior	15.45 (3.01)	14.75 (3.65)	15.74 (2.66)	3.341	0.001
Life appreciation behavior	18.72 (4.30)	16.61 (5.09)	19.58 (3.61)	7.260	<0.001

M (S.D.), median (standard deviation); SRSS, self-rating sleep scale.

### Psychological state and health behaviors in type D personality and non-type D personality groups

Regarding psychological stress and lifestyle behaviors, participants with type D personality demonstrated significantly higher levels of physical stress and stress index, along with significantly lower stress tolerance, compared with those without type D personality (*P* < 0.05). Additionally, participants with type D personality had significantly higher SRSS scores, indicating poorer sleep quality (*P* < 0.001). In terms of health behaviors, participants with type D personality scored significantly lower in exercise behavior, regular lifestyle behavior, dietary behavior, health responsibility behavior, stress management behavior, and life appreciation behavior (all *P* < 0.005) compared with their non-type D counterparts. No significant differences in other variables were identified between the two groups (Table 2).

### Vascular health in type D personality and non-type D personality groups

Regarding vascular health, the scores of peripheral vascular elasticity and overall vascular health in the type D personality group were significantly lower than those in the non-type D personality group (Table 3).

**Table 3:** Participants' vascular health in type D personality and non-type D personality groups.

Variables	Total (*n* = 645)	Type D (*n* = 186)	Non-type D (*n* = 459)	*Z* value	*P* value
Arterial elasticity, M (IQR)	79.00 (36)	80 (34)	79 (37)	−0.291	0.771
Peripheral vascular elasticity, M (IQR)	60 (20)	58 (20)	62 (17)	−2.090	0.037
Vascular health, M (IQR)	90 (34)	85 (32)	92 (8)	−7.685	<0.001
Waveform a, M (IQR)	240 (170)	240 (20)	240 (10)	−2.254	0.024
Waveform b, M (IQR)	272.75 (35.21)	268.85 (42.97)	273.39 (33.02)	−1.347	0.178
Waveform c, M (IQR)	39.25 (26.02)	36.94 (19.56)	44.21 (26.71)	−3.136	0.002

M (IQR), median (interquartile range).

### Multivariate regression analysis of vascular health by type D personality classification

Multivariate linear regression analysis was conducted to examine the association between vascular health and type D personality, in which vascular health score was taken as the dependent variable into account. Independent variables that exhibited significant differences in univariate analysis were included in the model. When type D personality was treated as a binary variable, it was significantly associated with poorer vascular health [*β* = −0.169, 95% confidence interval (CI): −4.001 to −1.483, *P* < 0.001]. Additionally, a higher AC was negatively associated with vascular health (*β* = −0.158, 95% CI: −0.188 to −0.031, *P* = 0.006), as were more severe sleep problems (*β* = −0.131, 95% CI: −0.301 to −0.076, *P* = 0.001). Conversely, greater stress tolerance (*β* = 0.208, 95% CI: 0.061–0.136, *P* < 0.001), more frequent engagement in exercise behavior (*β* = 0.084, 95% CI: 0.018–0.406, *P* = 0.032), and stronger stress management behavior (*β* = 0.233, 95% CI: 0.349–0.794, *P* < 0.001) were positively associated with better vascular health status (Table 4).

**Table 4:** Multivariate regression analysis of vascular health by type D personality classification.

Variables	Unstandardized B	SE	Standardized *β*	Test value	*P* value	95%CI
Type D	−2.742	0.641	−0.169	−4.276	<0.001	−4.001 to −1.483
Covariates						
Gender	−0.869	0.724	−0.046	−1.201	0.230	−2.291−0.552
BMI	0.057	0.118	0.027	0.484	0.629	−0.174−0.288
AC	−0.110	0.040	−0.158	−2.742	0.006	−0.188 to −0.031
SBP	−0.052	0.031	−0.082	−1.688	0.092	−0.113 to 0.009
DBP	0.003	0.039	0.003	0.076	0.940	−0.074 to 0.08
Pulse	−0.053	0.030	−0.072	−1.775	0.076	−0.111 to 0.006
Stress index	−0.030	0.038	−0.028	−0.773	0.440	−0.105 to 0.046
Stress tolerance	0.099	0.019	0.208	5.186	<0.001	0.061 to 0.136
Exercise behavior	0.212	0.099	0.084	2.148	0.032	0.018 to 0.406
Regular lifestyle behavior	−0.142	0.154	−0.049	−0.925	0.355	−0.444 to 0.16
Dietary behavior	−0.126	0.118	−0.055	−1.068	0.286	−0.358 to 0.106
Health hazard behavior	−0.175	0.214	−0.029	−0.819	0.413	−0.595 to 0.245
Health responsibility behavior	−0.122	0.095	−0.057	−1.283	0.200	−0.309 to 0.065
Interpersonal relationship behavior	0.087	0.074	0.053	1.180	0.239	−0.058 to 0.233
Stress management behavior	0.571	0.113	0.233	5.038	<0.001	0.349 to 0.794
Life appreciation behavior	−0.058	0.085	−0.034	−0.678	0.498	−0.224 to 0.109
SRSS	−0.188	0.057	−0.131	−3.283	0.001	−0.301 to −0.076

BMI, body mass index; AC, abdominal circumference; SBP, systolic blood pressure; DBP, diastolic blood pressure; SRSS, self-rating sleep scale.

### Multivariate regression analysis of vascular health: main effects and interaction of NA and SI

When type D personality was regarded as a continuous variable of NA and SI dimensions, the analysis revealed that NA was independently associated with poorer vascular health (*β* = −0.240, 95% CI: −0.569 to −0.049, *P* = 0.020). In contrast, SI and the interaction term between NA and SI exhibited no significant associations with vascular health. Among the covariates, AC (*β* = −0.171, 95% CI: −0.198 to −0.039, *P* = 0.004), pulse rate (*β* = −0.093, 95% CI: −0.127 to −0.010, *P* = 0.021), and sleep problems as measured by the SRSS score (*β* = −0.155, 95% CI: −0.336 to −0.110, *P* < 0.001) were negatively associated with vascular health. In contrast, higher stress tolerance (*β* = 0.211, 95% CI: 0.062–0.139, *P* < 0.001), more frequent exercise behavior (*β* = 0.078, 95% CI: 0.001–0.192, *P* = 0.048), and stronger stress management behavior (*β* = 0.226, 95% CI: 0.328–0.780, *P* < 0.001) were positively associated with better vascular health (Table 5).

**Table 5:** Multivariate regression analysis of vascular health with the main and interaction effects of NA and SI.

Variables	Unstandardized B	SE	Standardized *β*	Test value	*P* value	95%CI
NA	−0.309	0.132	−0.240	−2.334	0.020	−0.569 to −0.049
SI	−0.169	0.120	−0.137	−1.408	0.160	−0.405 to 0.067
NA*SI	0.010	0.007	0.231	1.456	0.146	−0.004 to 0.024
Covariates						
Gender	−0.929	0.735	−0.049	−1.263	0.207	−2.373 to 0.515
BMI	0.089	0.119	0.041	0.746	0.456	−0.145 to 0.322
AC	−0.119	0.041	−0.171	−2.922	0.004	−0.198 to −0.039
SBP	−0.058	0.032	−0.091	−1.819	0.069	−0.121 to 0.005
DBP	−0.003	0.040	−0.004	−0.084	0.933	−0.082 to 0.075
Pulse	−0.068	0.030	−0.093	−2.306	0.021	−0.127 to −0.010
Stress index	−0.036	0.039	−0.035	−0.931	0.352	−0.113 to 0.040
Stress tolerance	0.100	0.019	0.211	5.170	<0.001	0.062 to 0.139
Exercise behavior	0.196	0.100	0.078	1.958	0.048	0.001 to 0.192
Regular lifestyle behavior	−0.169	0.155	−0.058	−1.090	0.276	−0.474 to 0.136
Dietary behavior	−0.114	0.122	−0.050	−0.936	0.350	−0.353 to 0.125
Health hazard behavior	−0.170	0.217	−0.028	−0.784	0.433	−0.596 to 0.256
Health responsibility behavior	−0.083	0.096	−0.039	−0.867	0.386	−0.272 to 0.105
Interpersonal relationship behavior	0.078	0.075	0.047	1.039	0.299	−0.069 to 0.225
Stress management behavior	0.554	0.115	0.226	4.818	<0.001	0.328 to 0.780
Life appreciation behavior	−0.038	0.088	−0.022	−0.427	0.670	−0.210 to 0.135
SRSS	−0.223	0.058	−0.155	−3.877	<0.001	−0.336 to −0.11

NA, negative affectivity; SI, social inhibition; BMI, body mass index; AC, Abdominal circumference; SBP, systolic blood pressure; DBP, diastolic blood pressure; SRSS, self-rating sleep scale.

## Discussion

Type D personality has been independently associated with coronary atherosclerotic plaque development and adverse cardiovascular events in middle-aged and elderly individuals with cardiovascular disease, as supported by numerous studies (3, 4, 8). However, its potential impact on vascular health during adolescence remains elusive. Given that vascular elasticity is a precursor to atherosclerotic plaque formation, investigating whether type D personality influences vascular health in adolescents is of clinical relevance. This study aimed to explore the relationship between type D personality and vascular health in adolescents. To our knowledge, it is the first to examine this association in this age group. Consistent with our hypothesis, type D personality, when analyzed as a dichotomous variable, emerged as an independent risk factor for poorer vascular health in adolescents. However, beyond the categorical type D effect, the NA dimension emerged as the sole continuous, independent predictor of reduced vascular health in our analysis. Neither the SI dimension nor its interaction with NA demonstrated an independent influence. These findings strongly suggest that the association observed using the categorical type D classification likely represents a proxy effect of the underlying NA dimension. Consequently, when NA exhibits a strong association with vascular health outcomes, even if SI shows no independent association, the role of type D personality in vascular health will be demonstrated. This suggests that NA may be a key component driving the relationship between type D personality and vascular function in adolescents. Therefore, these findings should be interpreted with caution and in conjunction with analyses treating the personality dimensions as continuous variables.

Among 645 adolescents in this study, 186 had type D personality, accounting for 28.84%, which is almost consistent with previous research (18). The results of multivariate regression analysis revealed that type D personality was negatively correlated with vascular health. The NA dimension could negatively affect vascular health. Participants with high NA typically suppress emotional expression and experience psychological stress (3). Chronic exposure to psychological stress in such cases may lead to the persistent activation of the sympathetic-adrenal-medullary system and the hypothalamic-pituitary-adrenal (HPA) axis. This results in elevated cardiovascular reactivity, vascular smooth muscle cell proliferation, and reduced vascular elasticity (19). Elevated levels of stress hormones, such as cortisol, antidiuretic hormone, and adrenocorticotropic hormone, further accelerate vascular aging (2). Additionally, enhanced negative emotions are associated with increased secretion of inflammatory cytokines, which can trigger inflammatory responses involving macrophage infiltration, foam cell rupture, and apoptosis, ultimately contributing to vascular stiffness and progression of atherosclerosis (8). The results of the present study indicated that AC negatively affected vascular health. Short KR et al.'s study demonstrated that abdominal obesity can lead to initial endothelial dysfunction and vascular stiffness (20). Similarly, Noori NM et al. (21) also reported a significant association between obesity and arterial stiffness in overweight children. Among physiological indicators, elevated pulse rate was associated with reduced vascular health. Hao Yankai et al. (22) pointed out that aerobic exercise could improve vascular elasticity by enhancing heart rate reserve, enhancing cardiac output, reducing resting heart rate, and restoring autonomic nervous system balance. Stress tolerance, reflecting an individual's physical and psychological resilience to external stressors, was positively associated with vascular health. This suggests that individuals with greater stress resilience are more likely to maintain better vascular function. Yu ZM et al. (23) found that rabbits exposed to chronic stress exhibited signs of physiological decline, including weight loss, poor coat condition, unhealthy behaviors, elevated serum cortisol levels, and reductions in vascular smooth muscle cells and elastic fibers, leading to impaired vascular elasticity (24). Collectively, these findings highlight abdominal obesity, elevated pulse rate, and prolonged stress exposure as key risk factors for diminished vascular elasticity. These insights highlight the importance of promoting regular physical activity, weight management, and effective stress-coping strategies among adolescents. School administrators and educators should play an active role in improving supportive environments that encourage healthy lifestyle practices to mitigate early vascular decline.

In this study, poor sleep was found to negatively affect vascular elasticity. Carrera A. et al. (9) demonstrated that chronic sleep disruption in adult male mice led to a continuous increase in systemic blood pressure from the eighth week of intervention. By the end of the study, mice subjected to sleep disturbance exhibited remarkable vascular changes, including disruption and fragmentation of elastic fibers, increased accumulation of foam cells and macrophages along the aortic wall, elevated plasma IL-6 level, and upregulation of aging markers. These findings suggest that prolonged poor sleep promotes vascular inflammation, endothelial dysfunction, mild hypertension, and structural deterioration of vascular elastic fibers, ultimately reducing vascular elasticity and contributing to the development of cardiovascular diseases. Similar conclusions have been previously presented in community-based studies (10). A large number of human and animal experiments have demonstrated that negative emotions and sleep disturbances are critical factors in the decline of vascular health. Therefore, university administrators and student affairs professionals should give greater attention to promote emotional well-being and improve sleep hygiene among students. This includes providing psychological counseling services to promote positive emotional states, as well as implementing policies that encourage regular sleep schedules, such as going to bed and waking up early, to increase sleep duration, conserve energy, and enhance daytime functioning. These measures not only support students' health and academic performance, but also contribute to the overall quality of talent cultivation in higher education institutions.

In terms of lifestyle, exercise behavior and stress management exert positive effects on vascular elasticity, suggesting that healthy behaviors contribute to improved vascular function. Exercise behavior typically involves engaging in 30–60 min of aerobic activity at least three times per week. Nettlefold et al. (9, 25) found that individuals who regularly exercised exhibited greater arterial elasticity compared with those with sedentary lifestyles. This effect may be attributed to the ability of physical activity to reduce oxidative stress and inflammation while enhancing nitric oxide availability, exerting a vascular protective effect (26). Using murine experiments, Wang Chen et al. (27) found that extracellular vesicles may mediate the protective role of exercise in vascular elasticity, although the precise mechanisms remain to be fully elucidated. Stress management behavior refers to the capacity to organize tasks effectively and alleviate stress through appropriate coping strategies. Individuals with strong stress management skills are better equipped to mitigate the adverse effects of prolonged stress exposure on vascular health, which was previously documented (28). In summary, a healthy lifestyle is conducive to vascular health. Currently, health-seeking behaviors among college students remain at a moderate level (29). University administrators should consider enhancing students' health literacy and promoting healthy behaviors by organizing targeted health promotion initiatives. These may include digital learning strategies, such as health knowledge competitions, lectures, and the use of multiple online platforms (e.g., WeChat, QQ, “Internet Plus” teaching platforms, and short video services) to enhance student engagement. In addition, integrating health literacy into professional curricula and employing peer influence can promote a culture of proactive health management, ultimately contributing to a more health-conscious and dynamic campus environment.

## Conclusions

In this study, adolescents with type D personalities exhibited generally reduced vascular elasticity, remarkable sleep disturbances, and lower levels of overall health-promoting behaviors. Chronically elevated NA emerged as the key independent predictor of impaired vascular elasticity within the type D personality construct. Neither SI nor its interaction with NA demonstrated an independent influence on this outcome. Future research should prioritize enhanced monitoring of vascular health among cases with type D personality, especially those with a high NA score, alongside the implementation of personalized health behavior interventions and emotional regulation strategies. Long-term follow-up will be essential to promote sustained improvements in health management, strengthen health literacy, elevate overall health status, and ultimately reduce the risk of cardiovascular diseases in both adolescent and adult populations.

## Data Availability

The original contributions presented in the study are included in the article/Supplementary Material, further inquiries can be directed to the corresponding author.
